# Knowledge, attitudes, and practices towards COVID-19 among college students in China: A systematic review and meta-analysis

**DOI:** 10.1371/journal.pone.0270038

**Published:** 2022-06-16

**Authors:** Ling Li, Fang Wang, Xiaoling Shui, Qian Liang, Jingyi He

**Affiliations:** 1 School of Nursing, Chengdu University of Traditional Chinese Medicine, Chengdu, Sichuan Province, China; 2 Nursing Department, Affiliated Hospital of Chengdu University of Traditional Chinese Medicine, Chengdu, Sichuan Province, China; Middle East Liver Diseases (MELD) Center, Tehran, Iran, ISLAMIC REPUBLIC OF IRAN

## Abstract

**Background:**

Since the outbreak of the respiratory infectious disease caused by the novel coronavirus in 2019, the COVID-19 epidemic has posed a serious threat to the life and safety of the public, and has also seriously affected the normal study and life of college students in China. Although a series of studies have been conducted on college students’ knowledge, attitudes and practices of COVID-19, the results vary widely. This study aimed to evaluate the pooled estimated level of knowledge, attitudes, and practices (KAP) about COVID-19 among college students in China.

**Methods:**

We conducted a comprehensive search on Scopus, ProQuest, PubMed, EMbase, Web of Science, the Cochrane Library, Chinese Biomedical Literature Database (CBM), China National Knowledge Infrastructure (CNKI), VIP Database and Wanfang Database up to 13 February 2022. We then assessed the quality of included studies using a checklist developed by the Joanna Briggs Institute (JBI) for cross-sectional studies and analyzed using STATA.15 after two researchers independently extracted relevant data and entered them into Microsoft Excel. Funnel plots and Egger’s regression tests were used to check for publication bias, and sensitivity analysis was performed to assess the robustness of the results. A random-effects model was used for the meta-analysis, on the basis of which subgroup analyses were performed by time of investigation (study period) and by gender and major of the subjects.

**Result:**

A total of 42 studies including 174,440 subjects were included in this review, and the quality of the included studies was mainly intermediate and advanced. The findings of the meta-analysis showed that the overall levels of Chinese college students’ knowledge, positive and negative attitudes, and practice of preventive measures towards COVID-19 were 74% (95%CI: 69%-79%), 84% (95%CI: 80%-88%), 31% (95%CI: 23%-38%) and 82% (95%CI: 77%-86%), respectively. The results of subgroup analysis showed that female and medical college students had higher levels of knowledge and practice on COVID-19.

**Conclusion:**

The study findings showed that the overall KAP level of college students in China included in the study was relatively optimistic. Influenced by gender, major and time, there were differences in the KAP level of college students. In order to promote the effective prophylaxis and control of pandemic, we recommend implementing targeted measures to improve the awareness rate of COVID-19-related knowledge among this group and the implementation rate of COVID-19 preventive measures among male and non-medical college students.

## Introduction

In late December 2019, a cluster of severe pneumonia patients of unknown cause was reported in Wuhan, China. In the testing of lower respiratory tract samples from 4 patients, a novel strain of coronavirus belonging to the same family of viruses that cause Middle East Respiratory Syndrome (MERS) and Severe Acute Respiratory Syndrome (SARS), as well as the four human coronaviruses related to the common cold, was isolated [1]. On January 30,2020, the World Health Organization (WHO) declared the novel coronavirus outbreak a public health emergency of international concern, and proposed to name the disease caused by the virus as COVID-19 on February 11 [2]. As of 10 March 2022, COVID-19 has spread to 225 countries and territories, with more than 6 million deaths [3].

According to existing studies, COVID-19 has the characteristics of strong infectivity, easy mutation, and general susceptibility of the population [4, 5]. Although targeted vaccines have been developed and widely used in the population, the duration of immunity obtained from vaccination is unclear [5]. Previous study [6] has shown that carrying out KAP surveys during the epidemic is conducive to understanding the public’s awareness of epidemic prevention and weak points of practice, so as to implement precise health education. Al Ahdab S [7] pointed out that good awareness, positive attitudes and qualified behaviors about COVID-19 are critical to the successful prophylaxis and control of the epidemic.

While there were existing studies on the knowledge, attitudes and practices (KAP) towards COVID-19 among college students in China, they were not comprehensive and the results obtained vary. A review article [8] evaluated public awareness of COVID-19 in China, but no meta-analysis was conducted. Another review article [9] evaluating knowledge, attitudes, and practices about COVID-19 among healthcare workers and the general population also did not perform any quantitative analysis. In view of the importance of individual cognition and behavior in the effective prophylaxis and control of the epidemic and the particularity of this group, it is necessary to conduct a systematic review and meta-analysis of KAP of this population to provide a pooled estimated proportion of KAP for COVID-19 among college students in China, and provide reference for relevant departments to carry out scientific prophylaxis and control during the pandemic. To our knowledge, this is the first systematic review and meta-analysis of college students’ knowledge, attitudes, and practices about COVID-19.

## Methods

This systematic review and meta-analysis was conducted in accordance with the ‘Preferred Reporting Items for Systematic Reviews and Meta-analyses’ (PRISMA) [10] (S2 File). The systematic review protocol has been registered on PROSPERO (CRD42022316375).

### Eligibility criteria

#### Inclusion criteria

All observational studies on KAP towards COVID-19 among college students in China were considered for this study, with no restrictions on the gender, health status of the subjects, and language, time, quality or geographic location of the study. However, only studies that were available in full text and reported the sample size of the study and data related to any one component of the KAP, or provided data from which required outcomes could be calculated, were included.

#### Exclusion criteria

We excluded certain studies that were conducted only on specific populations, such as medical students. In addition, review articles, studies with confused or potentially erroneous data, studies for which raw data could not be extracted or transformed, and studies reporting perception on coronaviruses other than COVID-19 would also be excluded.

### Information sources, search strategy and study selection

The two researchers conducted systematic searches in Scopus, ProQuest, PubMed, EMbase, Web of Science, the Cochrane Library, Chinese Biomedical Literature Database (CBM, http://www.sinomed.ac.cn/index.jsp), China National Knowledge Infrastructure (CNKI, https://www.cnki.net/), VIP Database (http://lib.cqvip.com/) and Wanfang Database (https://new.wanfangdata.com.cn/index.html). In addition, other studies including grey literature were searched through Baidu Xueshu and Google Scholar. The main keywords for the search strategy included “Covid-19”, “2019-nCoV”, “SARS-CoV-2”, “Severe Acute Respiratory Syndrome Coronavirus 2”, “knowledge”, “perception”, “awareness”, “consciousness”, “attitude”, “practice”, “action” and “KAP”. Search strategies for PubMed and CNKI databases are provided in S2 File. The preliminary search, which was carried out on 1 February 2022 and updated on 13 February. The retrieval results were stored and managed through Endnote software.

After screening out duplicate articles, two researchers independently performed preliminary screening of the remaining articles by reading the titles and abstracts according to pre-established criteria. When the title and abstract were insufficient to judge whether to exclude an article, the researcher would make the choice after reading the full text. Any disagreements would be resolved through discussions and consultations with a third researcher.

### Study outcomes

This study involved three main outcomes of KAP associated with COVID-19 among college students in China.

#### Knowledge

Main symptoms, routes of transmission, susceptible population, incubation period, knowledge or awareness of preventing COVID-19.

#### Attitudes

Attitudes towards managing or controlling of COVID-19, and fears or worrisome about COVID-19.

#### Practices

Measures such as wear masks, maintain social distance, avoid crowded places/social activities and hand hygiene.

### Data extraction

The relevant data were independently extracted and entered into an Excel spreadsheet template by two researchers. Include: first author, year and month of study, study area, study design, sample size, demographics of study subjects, and study results related to any component of KAP. (The mastery and implementation of knowledge and practices by participants in each study was obtained by calculating the overall average proportion of each component of KAP.) In addition, the proportion of specific content in each component of KAP was also extracted from the included studies. Any disagreements during extraction were resolved through discussion and negotiation. When necessary, we would contact study authors for missing data.

### Quality assessment

The quality of included studies was independently assessed by two researchers using the JBI Quality Assessment Checklist [11], which consists of eight questions, namely: (1) Were the inclusion criteria for study subjects clear?, (2) Were the research objects and places described in detail?, (3) Was the assessment method of exposure factors valid and credible?, (4) Was the assessment method of health problems objective and standard?, (5) Were the confounding factors clearly defined?, (6) Were there any measures to control confounders?, (7) Were the outcomes measured in a valid and credible way?, and (8) Was the statistical analysis appropriate?. Each question has three options of “yes, no, not clear”, each of which accounts for 1 point, 0 point, and 0.5 point. After summarizing the scores obtained for each question, studies were considered to have a high risk of bias and low quality if the total score was less than 3 points; moderate risk of bias and moderate quality if the total score was 3 to <6 points; low risk of bias and high quality if the total score was 6–8 points [12]. Differences were resolved through discussions and consultations with a third researcher.

### Statistical analysis

Descriptive analysis was used in most sections to report relevant content, and the statistical software package STATA.15 was used to analyze quantitative data extracted from each study. Funnel plots and Egger’s regression test were used to check for publication bias, with P <0.05 considered to exist potential publication bias [13]. The Cochrane Q statistic was used to test for heterogeneity between studies, and the heterogeneity was quantified by the I^2^ value, where 25%, 50%, and 75% indicated low, moderate, and high heterogeneity, respectively [14]. As heterogeneity was found to be greater than 50%, we used the random effect model for the analysis. Subgroup analyses were also performed by study period (every 3 months as a subgroup), gender and major of subjects to explore sources of heterogeneity. To assess the robustness of the findings, we also performed a sensitivity analysis [15]. The final results were reported as pooled proportion and 95% confidence interval for COVID-19 KAP and were presented in forest plot.

## Results

### Search outcomes

**Fig 1:**
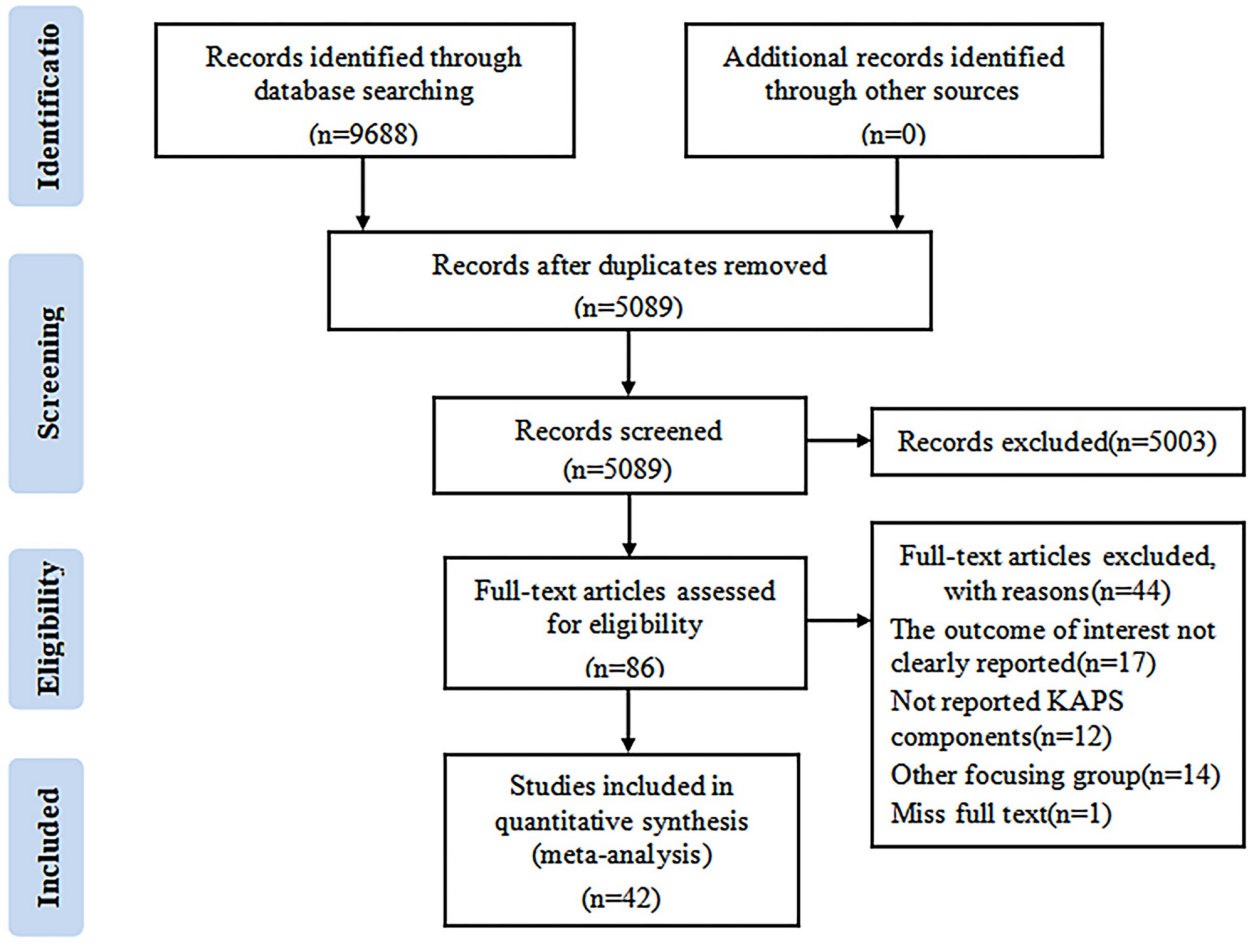
PRISMA 2009 flow diagram.

A total of 9,688 articles were retrieved from the selected database. After excluding 4,599 duplicate articles, another 5,047 articles were excluded according to the inclusion and exclusion criteria on the basis of reading the title, abstract and full text. Finally, 42 articles were included for qualitative and quantitative analysis, including 174,804 participants [16–57]. The PRISMA flow chart for the study selection process is presented in Fig 1.

### Characteristics of included studies

A total of 42 studies encompassing 174,440 participants were included, most of which were conducted in 2020. Except for the 4 studies that did not elucidate the gender characteristics of the participants, in the remaining 38 studies, 60.63% of the participants were female.

All studies employed a cross-sectional design, and data were collected primarily through online questionnaires. Nine studies reported all three components of KAP, and the remaining 33 reported only two components or one component of KAP. The sample size of each study varied from 134 to 44446. For more information on included studies and participants characteristics, see S1 File.

### Quality assessment of included studies

The overall average score of the included studies was 4.74 according to the JBI quality assessment checklist. Among them, 16 studies (38.1%) were rated as good quality (score ≥6) and 26 (61.9%) as moderate quality (score 3–5). All studies failed to score on questions 5 and 6, which were related to the failure to identify and deal with confounding factors in the research process. (See S2 File for details).

### Synthesis of results

**Fig 2:**
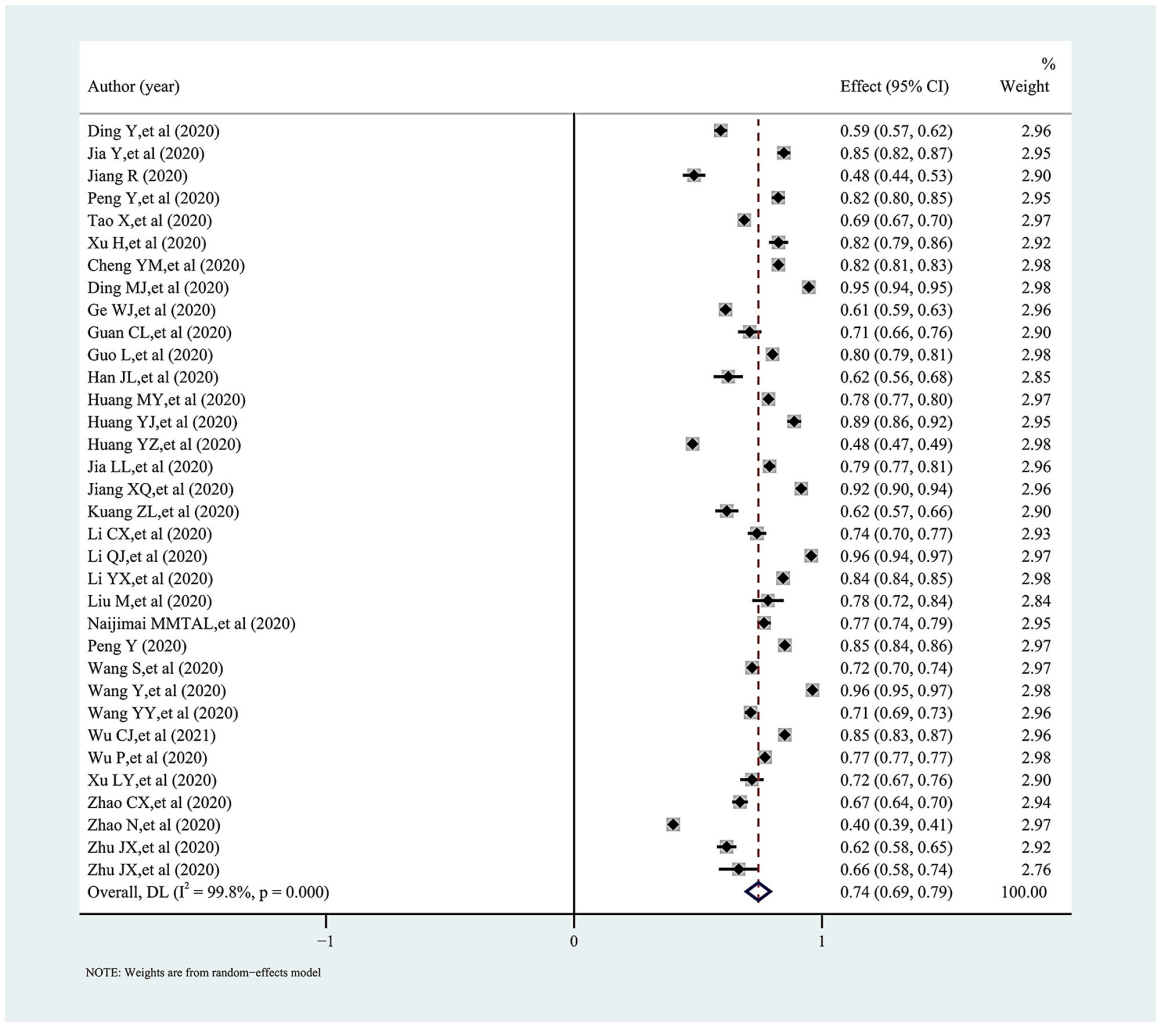
Forest plot showing the overall proportion of knowledge about COVID-19 among study subjects.

**Fig 3:**
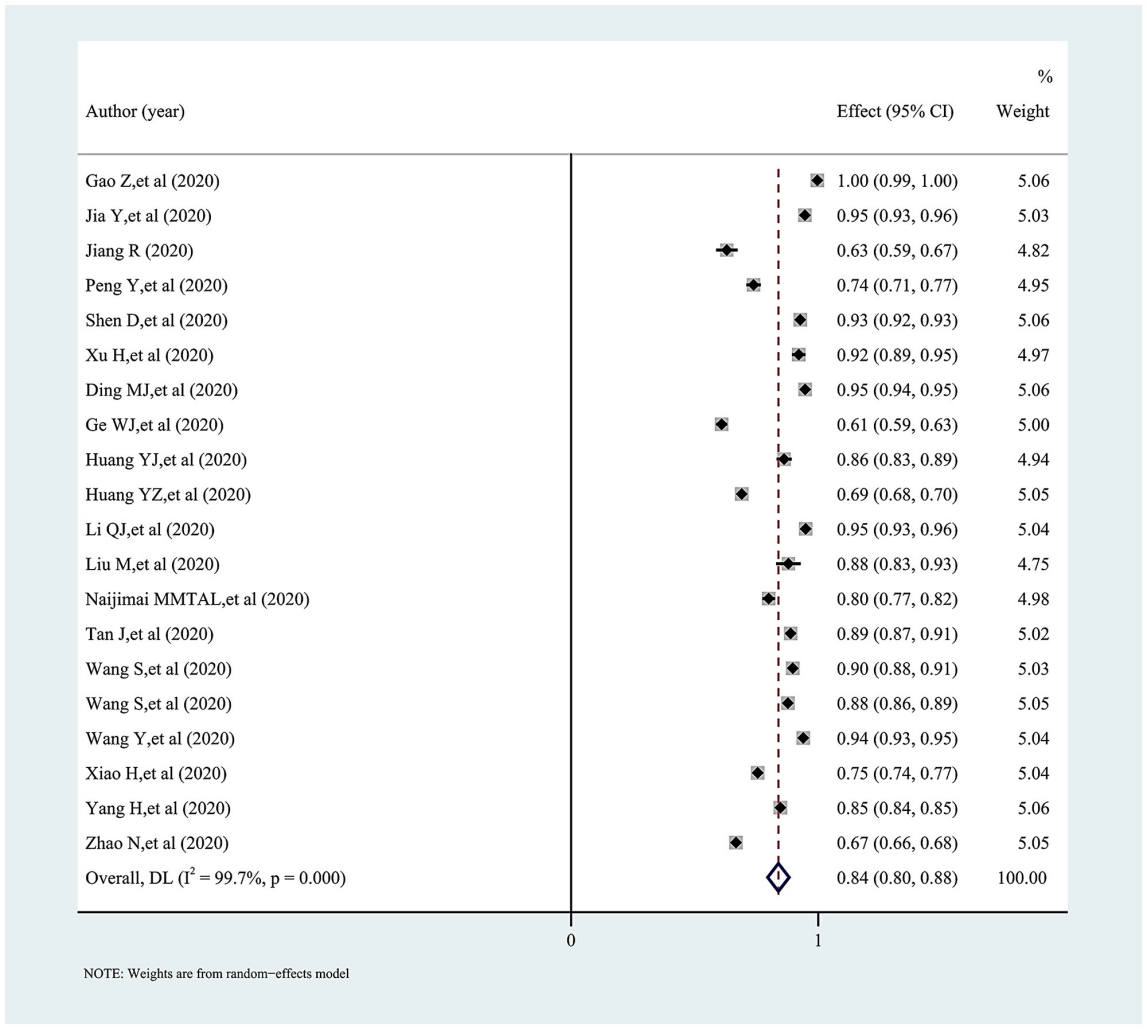
Forest plot showing the pooled proportion of positive attitudes towards COVID-19 among study participants.

**Fig 4:**
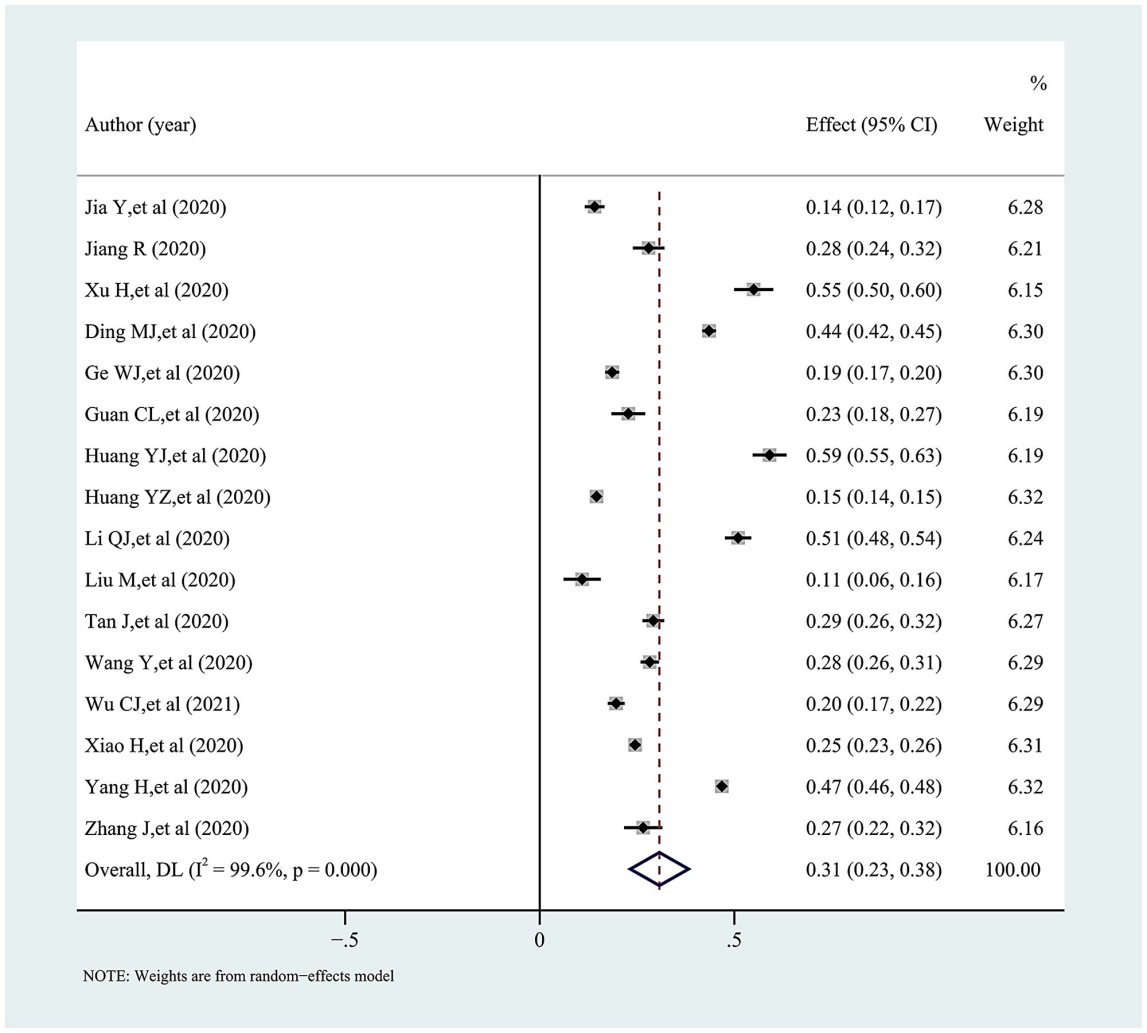
Forest plot showing the pooled proportion of negative attitudes towards COVID-19 among study participants.

**Fig 5:**
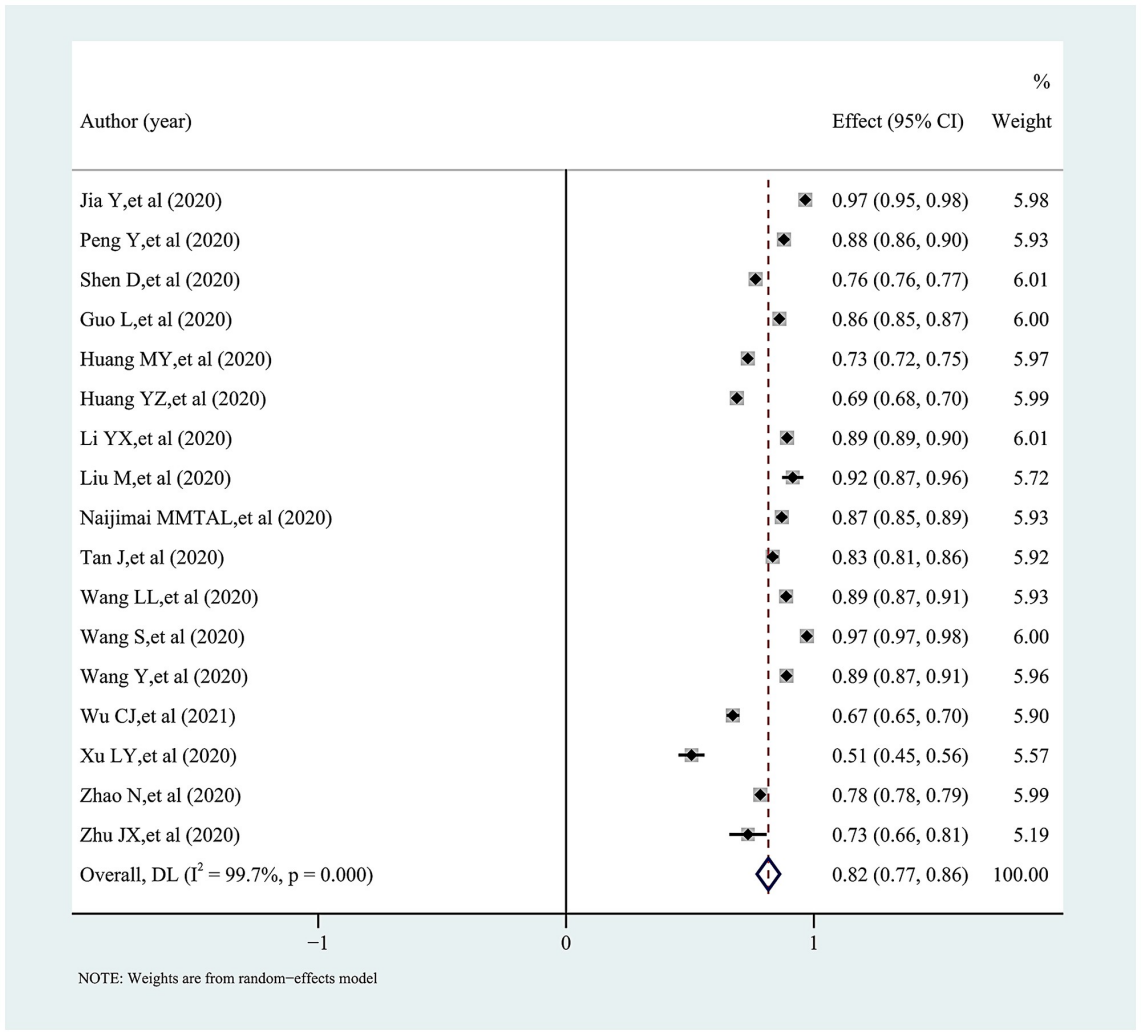
Forest plot showing the pooled proportion of using specific measures to fight COVID-19 among study subjects.

The pooled proportion and 95% confidence interval of knowledge, attitudes, and practices towards COVID-19 among college students in China is presented in a forest plot (Figs 2–5). Among them, the pooled proportion of knowledge about COVID-19 was 74% (95%CI: 69%-79%, I^2^ = 99.8%), the pooled proportions of positive and negative attitudes towards COVID-19 were 84% (95%CI: 80–88%, I^2^ = 99.7%) versus 31% (95%CI: 23%-38%, I^2^ = 99.6%), and the pooled proportion for COVID-19 practice level was 82% (95%CI: 77%-86%, I^2^ = 99.7%).

In addition, further analysis was carried out on the specific content of each component of KAP. The results showed that the pooled awareness proportions of Chinese college students about the main symptoms, routes of transmission, susceptible population, incubation period and preventive measures of COVID-19 were 80%, 73%, 43%, 87%, and 70%, respectively. The pooled proportions of practice for specific precautions against COVID-19 (wearing masks, washing hands, and social distancing) were 90%, 84%, and 87%, respectively. (See S2 File for details).

### Publication bias analysis

**Fig 6:**
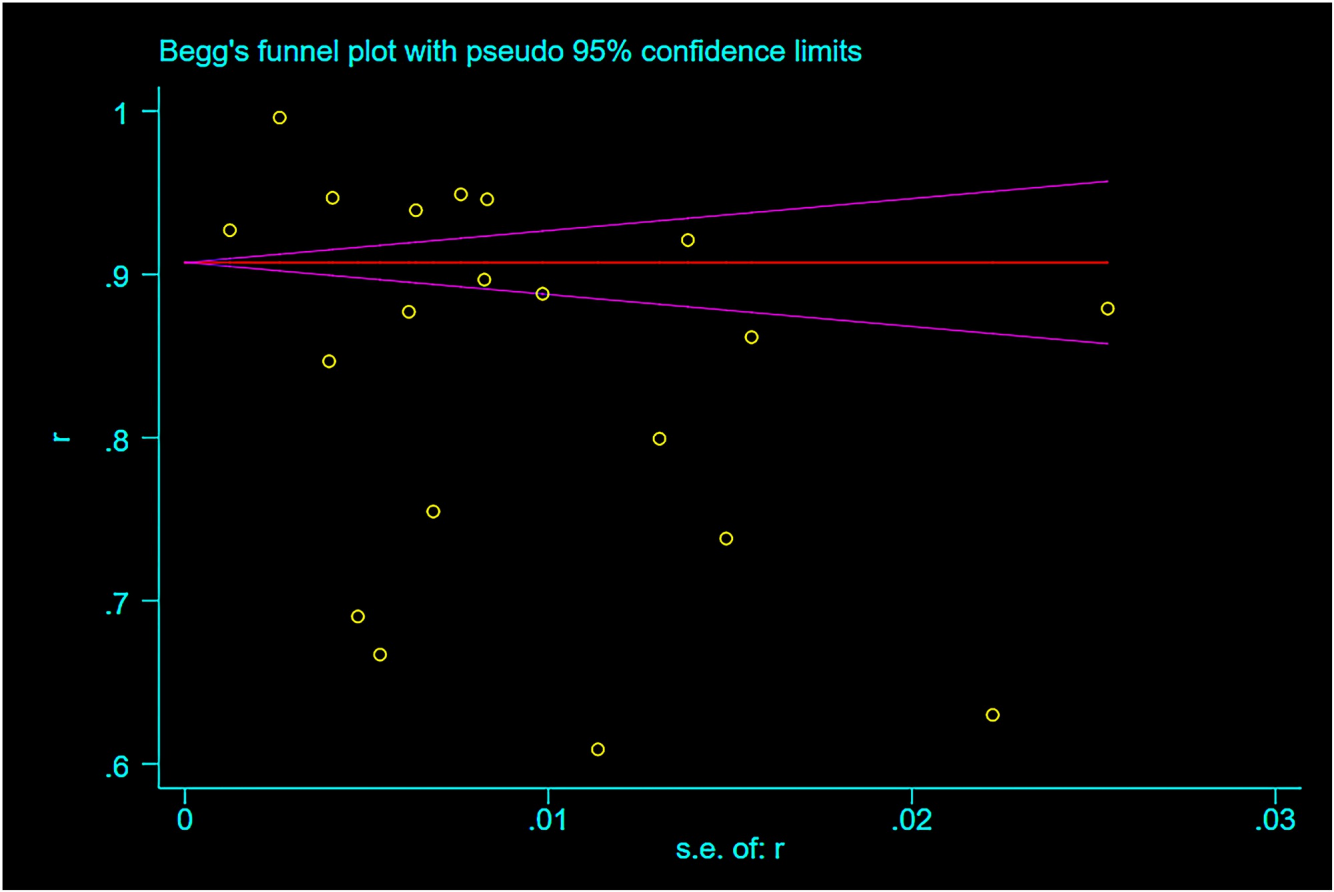
Begg’s funnel plot checking the publication bias of proportion of positive attitudes about COVID-19 among study subjects.

Publication bias was evaluated by funnel plot and Egger’s regression test. The funnel plot and Egger’s regression test results indicated that: there was publication bias among the studies on positive attitudes towards COVID-19 among college students in China (P = 0.043 <0.05) (Fig 6). In addition, the research points of the other three parts were basically symmetrical in the funnel plot, and the results calculated by Egger linear regression (P = 0.333, P = 0.274, P = 0.867) also indicated that there was no significant publication bias. The result was corrected by the trim-and-fill method, and the result [83%, 95%CI: 77.6%-88.5%, P<0.001] was not significantly different from the value before correction [84%, 95%CI: 80%-88%, P<0.001], indicating that the pooled proportion of positive attitudes towards COVID-19 among college students in China was less affected by publication bias.

### Sensitivity analysis

The robustness of the results was assessed using a leave-out-one sensitivity analysis, which showed that stepwise exclusion of each study did not result in any significant changes in the pooled proportion for knowledge, positive and negative attitudes, and level of practice. (See S2 File for details).

### Subgroup analysis

The results of subgroup analysis based on the gender and major of participants showed that women had relatively higher knowledge (75%, 95%CI: 63%-86% vs 72%, 95%CI: 61%-82%) and practice (79%, 95%CI: 72%-85% vs 76%, 95%CI: 67%-85%) compared with men, with no significant difference in attitude towards COVID-19 (79%, 95%CI: 70%-88% vs 79%, 95%CI: 68%-89%). Compared with non-medical college students, medical college students had a higher level of knowledge towards COVID-19 (75%, 95%CI: 57%-92% vs 66%, 95%CI: 55%-78%), more positive attitude (76%, 95%CI: 65%-87% vs 73%, 95%CI: 60%-87%) and higher level of practice (79%, 95%CI: 66%-92% vs 75%, 95%CI: 66%-85%).

The results of subgroup analysis based on the study period showed that over time, the pooled proportions of the mastery and practice of COVID-19-related knowledge and preventive measures among college students in China showed an upward and downward trend, respectively. But there was no noticeable change in their attitudes towards COVID-19 (Table 1). Subgroup analyses for negative attitudes were not performed because of the relatively small number of studies reporting specific data on gender and major among all studies on negative attitudes, and the study period focused on the first three months.

**Table 1:** Subgroup analysis based on gender and major.

Subgroup analysis based on gender and major				
Outcome	Study characteristics	No of studies	Estimates score (%) (95%CI),p-value	I^2^
**Knowledge**	**gender**	10		
	male		72 (61–82) <0.001	99.4%
	female		75 (63–86) <0.001	99.8%
	**majors**	9		
	medical students		75 (57–92) <0.001	99.8%
	non-medical students		66 (55–78) <0.001	99.6%
	**Study period**			
	January-March, 2020	22	73 (67–80) <0.001	99.8%
	April-June, 2020	4	75 (64–86) <0.001	97.8%
	July 2020 and beyond	3	75 (66–84) <0.001	98.9%
**Positive attitude**	**gender**	7		
	male		79 (70–88) <0.001	99.1%
	female		79 (68–89) <0.001	99.7%
	**majors**	6		
	medical students		76 (65–87) <0.001	99.8%
	non-medical students		73 (60–87) <0.001	99.8%
	**Study period**			
	January-March, 2020	16	83 (78–88) <0.001	99.8%
	April-June, 2020	2	83 (77–89) 0.002	89.3%
**Practice**	**gender**	8		
	male		76 (67–85) <0.001	99.6%
	female		79 (72–85) <0.001	99.7%
	**majors**	5		
	medical students		79 (66–92) <0.001	99.8%
	non-medical students		75 (66–85) <0.001	99.6%
	**Study period**			
	January-March, 2020	10	82 (76–87) <0.001	99.7%
	April-June, 2020	3	76 (61–91) <0.001	98.9%

## Discussion

Although China has long entered the stage of normalizing the prophylaxis and control of the COVID-19 epidemic, clustered epidemic events on campus still occured from time to time. As a global pandemic that the most extensive to afflict humanity in a century [58], the prophylaxis and control of the COVID-19 is still a huge challenge of facing the world. As a special group with high educational level and high activity, college students’ epidemic prevention literacy level is not only related to their own health status, but also affects the cognition and behavior of the people around them to a certain extent. In this context, assessing the overall proportion of knowledge, attitudes, and practices towards COVID-19 among college students in China may help relevant departments to improve prophylaxis and control strategies for the COVID-19 epidemic.

The findings of this meta-analysis showed that the pooled proportion of college students’ knowledge of COVID-19 was 74%, which is similar to the findings reported by Aynalem YA et al. [59]. The results of subgroup analysis showed that the pooled proportion of Chinese college students’ mastery of COVID-19-related knowledge had gradually increased over time, and female and medical college students had a higher level of knowledge towards COVID-19. Similar findings were also reported in South Korea [60] and the United Arab Emirates [61]. These differences may be caused by the following reasons: in terms of time, less was known about the new disease at the beginning of the outbreak, while the gradual release of relevant information and guidelines later provided more knowledge about the disease. In terms of gender, on the one hand, the proportion of women in most studies is higher; on the other hand, it may be related to men’s risk-taking tendency and vulnerability to peer influence [62]. In terms of majors, on the one hand, medical students have more time and opportunities to contact health information and receive medical-related education, thereby developing a stronger sense of self-protection; on the other hand, medical students may be inclined to acquire and update their knowledge of COVID-19 from academic media and research articles. Although college students’ overall awareness of COVID-19-related knowledge was relatively optimistic, their awareness of specific content such as “susceptible population, routes of transmission and preventive measures” still needs to be improved. This also suggests that schools and relevant departments should carry out targeted publicity and education strategies in time.

The pooled proportion of subjects with positive attitudes about COVID-19 in this study was 84%, which was higher than the reported results of two studies conducted in Ethiopia (67.2%) [63] and Japan (68.5%) [64]. This might be related to the effectiveness of China’s control of COVID-19 as well as the concerted prevention and control efforts implemented across the country [65]. However, this study showed that the pooled proportion of participants who were worried or fearful about COVID-19 was 31%, which is much higher than the results reported by He LP et al. [66] during the SARS epidemic (12.5%). The reasons may be related to the long duration and wide spread of COVID-19, as well as the repeated closure measures taken by colleges and universities to avoid campus transmission. Relevant departments need to pay attention to the psychological status of this group and provide supportive measures to improve the psychological coping and resilience of college students during and following COVID-19 [67].

Furthermore, the pooled proportion for COVID-19 practice level was 82%, which was slightly lower than the findings of Yang K et al. [68] (84.4%). The results of subgroup analysis showed that female and medical college students had better practice level for COVID-19. However, the pooled proportion of Chinese college students’ practice of preventive measures against COVID-19 had a decreasing trend, which might be related to their reduced risk perception of the disease [69, 70]. As far as the pooled results were concerned, both in terms of overall level and specific measures, the implementation of COVID-19-related preventive measures among college students in China was relatively optimistic.

In this study, we observed a significant heterogeneity (I^2^>99%) in the pooled proportions of KAP components. To find the sources of heterogeneity, we used subgroup analysis and “a leave-out-one” sensitivity analysis were conducted to further assess relevant studies. The results showed that the pooled results of KAP components were robust and not influenced by a single study. Therefore, we considered that the high heterogeneity might be caused by differences in the original study sample size, questionnaire content, definition of results, and measurement methods.

### Strength and limitation of this study

About the advantages of this study: First, a systematic search was performed in multiple representative databases. Second: the quality of the included studies were mainly at the middle and high levels, and included most of the regions in China, which had a good representativeness. Third: to our knowledge, this is the first systematic review of KAP for Chinese college students on COVID-19, and the findings provided some meaningful data.

Limitations of this study: First, the heterogeneity among studies was high. Despite a series of subgroup analyses, the sources of heterogeneity could not be determined. Second, the studies included all used a cross-sectional design, which inevitably has inherent shortcomings of this design. Third, the original research lacks uniform measurement tools and operational definitions.

## Conclusion

This study showed that the overall levels of components of KAP related to COVID-19 were higher among college students in China. In terms of knowledge and practices, female and medical college students had better mastery and implementation. Although the overall situation is optimistic, in terms of knowledge, corresponding measures need to be implemented to increase the awareness rate of COVID-19 in this group.

## Supporting information

S1 Checklist PRISMA checklist. (DOC)

S1 File Relevant data extracted from reported studies. (XLSX)

S2 File (DOC)
